# Impact of Renal Function on Serum Free Light Chain Interpretation: A Comprehensive Review and Call for Stratified Reference Intervals

**DOI:** 10.7759/cureus.108866

**Published:** 2026-05-14

**Authors:** Sushant Dhanavade, Anshuli Vispute

**Affiliations:** 1 Department of Biochemistry, Krishna Institute of Medical Sciences, Krishna Vishwa Vidyapeeth (Deemed to be University), Karad, IND; 2 Department of Pathology, Krishna Institute of Medical Sciences, Krishna Vishwa Vidyapeeth (Deemed to be University), Karad, IND

**Keywords:** chronic kidney disease, diagnostic accuracy, elderly, free light chains, laboratory standardization, monoclonal gammopathy, reference intervals, renal impairment

## Abstract

The serum free light chain (FLC) assay has transformed the diagnosis and monitoring of plasma cell dyscrasias, yet its interpretation is significantly complicated by renal impairment. This comprehensive review, synthesizing evidence from 24 studies identified through a systematic search of PubMed, Scopus, and Web of Science (from inception to September 2023), examines the relationship between renal function and FLC metabolism, evaluates limitations in current reference interval application, and proposes strategies for improving diagnostic accuracy through renal function-stratified interpretation. The literature consistently demonstrates a strong inverse correlation between estimated glomerular filtration rate (eGFR) and serum FLC concentrations. Current manufacturer-derived reference intervals, established in young, healthy populations, lead to high false-positive rates in elderly patients with renal impairment, triggering unnecessary investigations. Studies proposing adjusted thresholds demonstrate significant potential for reducing false-positive interpretations without compromising sensitivity for detecting true monoclonal gammopathies. We advocate for the widespread adoption of eGFR-stratified reference intervals to reduce unnecessary investigations, decrease healthcare costs, and improve patient care.

## Introduction and background

The development of serum free light chain (FLC) assays represents one of the most significant advances in hematological diagnostics of the 21st century. First introduced in 2001, these assays revolutionized the detection and monitoring of plasma cell dyscrasias, including monoclonal gammopathy of undetermined significance (MGUS), multiple myeloma, and light chain amyloidosis [1]. By measuring circulating unbound immunoglobulin light chains, FLC assays provide sensitive detection of clonal plasma cell populations, with the kappa/lambda ratio serving as a specific indicator of monoclonality [2].

Despite their diagnostic power, FLC assays present significant interpretative challenges, particularly in populations with impaired renal function. Light chains are low-molecular-weight proteins (approximately 22-25 kDa) that are freely filtered by the glomerulus and subsequently reabsorbed and catabolized by proximal tubular cells [3]. This renal clearance pathway means that serum FLC concentrations are intrinsically linked to glomerular filtration rate (GFR), creating a fundamental physiological relationship that complicates clinical interpretation.

The problem is particularly acute in elderly populations, where age-related decline in renal function is nearly universal. Epidemiological studies indicate that approximately 40% of individuals over 70 years have an estimated GFR (eGFR) below 60 mL/min/1.73 m², with even higher prevalence in hospitalized and comorbid populations [4]. In these patients, the application of manufacturer-derived reference intervals-established in young, healthy individuals with normal renal function-frequently leads to false-positive interpretations, where elevated polyclonal FLCs secondary to reduced renal clearance are misinterpreted as evidence of monoclonal gammopathy [5].

The clinical consequences of these false positives are substantial and far-reaching. Patients may undergo unnecessary specialist hematology consultations, extensive laboratory evaluations, skeletal surveys, advanced imaging studies, and occasionally invasive bone marrow biopsies [6]. Beyond the direct healthcare costs, these investigations create significant patient anxiety, potential procedural risks, and delays in addressing alternative diagnoses. Furthermore, in patients with true monoclonal gammopathies and concurrent renal impairment, distinguishing disease progression from renal function fluctuations becomes diagnostically challenging. This comprehensive review aims to synthesize current knowledge on the relationship between renal function and serum FLC concentrations, critically evaluate the limitations of existing reference intervals, and propose evidence-based strategies for improving diagnostic accuracy through renal function-stratified interpretation.

## Review

Search strategy and selection of studies

**Figure 1:**
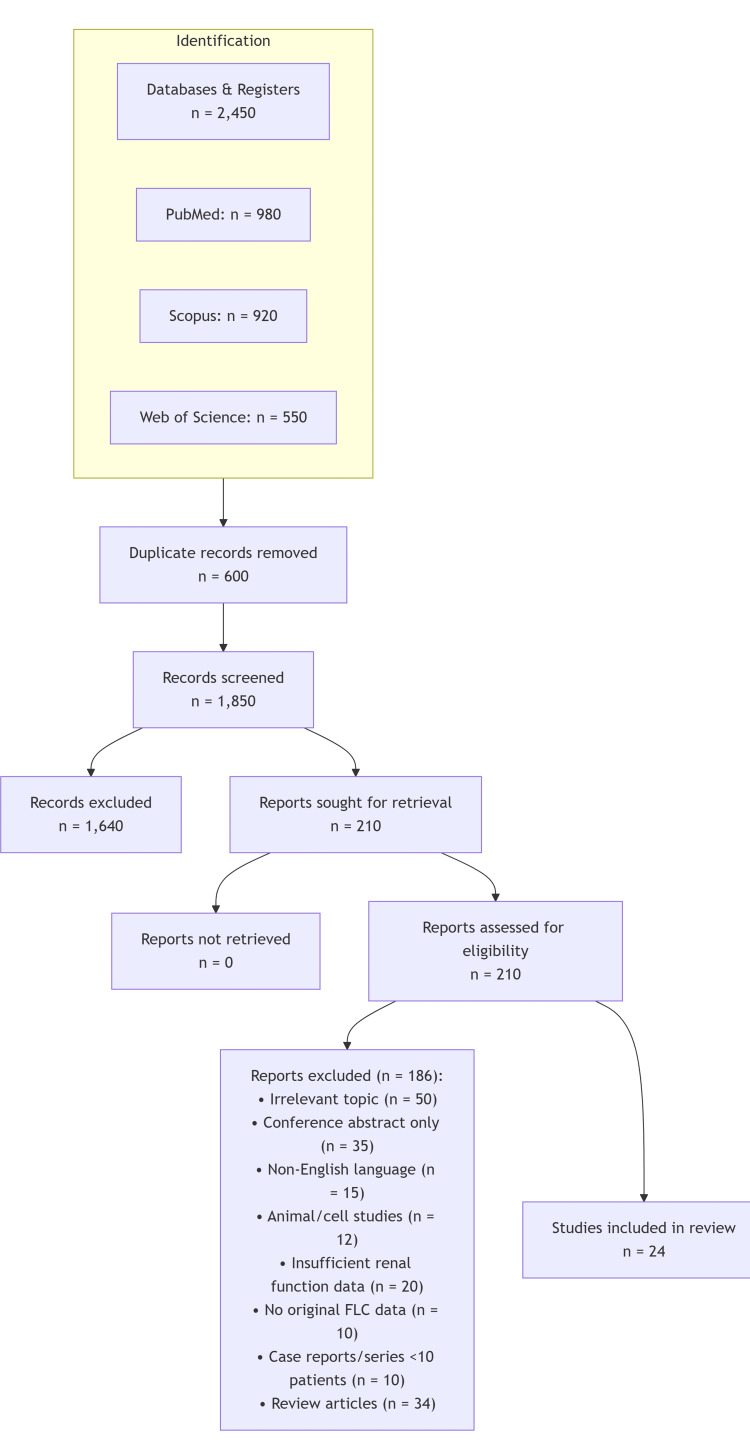
PRISMA Flow Diagram for Study Selection Flow diagram illustrating the systematic identification, screening, eligibility assessment, and inclusion process for studies in this comprehensive review. A total of 24 studies were selected for qualitative synthesis following the removal of duplicates and application of exclusion criteria. Databases searched: PubMed, Scopus, and Web of Science. FLC: free light chain; PRISMA: Preferred Reporting Items for Systematic reviews and Meta-Analyses

A systematic literature search was conducted to identify relevant studies on the relationship between renal function and serum FLC interpretation. Electronic databases searched included PubMed, Scopus, and Web of Science. The search strategy utilized a combination of Medical Subject Headings (MeSH) terms and keywords: (‘free light chains’ OR ‘immunoglobulin light chains’) AND (‘renal function’ OR ‘glomerular filtration rate’ OR ‘kidney disease’ OR ‘renal impairment’) AND (‘reference interval’ OR ‘reference range’ OR ‘interpretation’ OR ‘diagnostic accuracy’). The search was conducted from database inception to September 2023, with no language restrictions applied initially, though only English-language articles were ultimately included. The screening process followed the Preferred Reporting Items for Systematic reviews and Meta-Analyses (PRISMA) 2020 guidelines. After removing duplicates, 1,850 records were screened by title and abstract. Of these, 210 full-text articles were assessed for eligibility. After applying exclusion criteria (irrelevant topic, conference abstracts, non-English language, animal/cell studies, insufficient renal function data, no original FLC data, case reports/series with <10 patients, and review articles), a total of 24 studies were included for qualitative synthesis in this review, as shown in Figure 1.

Physiological basis of FLC clearance

Normal Renal Handling of Light Chains

Understanding FLC interpretation requires an appreciation of their normal renal metabolism. Immunoglobulin light chains exist in two primary forms: bound to heavy chains in intact immunoglobulins and as FLCs. While intact immunoglobulins are too large for glomerular filtration (approximately 150 kDa), FLCs readily pass through the glomerular basement membrane due to their smaller size (22-25 kDa) [7].

Renal Clearance of FLCs Involves Three Sequential Processes

Glomerular filtration: Approximately 90% of circulating FLCs are filtered at the glomerulus, with filtration efficiency influenced by molecular charge and configuration. Kappa light chains, being more cationic, exhibit slightly higher filtration rates than their anionic lambda counterparts [8].

Proximal tubular reabsorption: Filtered FLCs are almost completely reabsorbed in the proximal tubule via receptor-mediated endocytosis through the megalin-cubilin complex. This process is saturable, with a maximum transport capacity (Tm) that can be exceeded at high FLC concentrations [9].

Intracellular catabolism: Once internalized, FLCs are transported to lysosomes and degraded into amino acids, which are returned to the circulation. Minimal intact FLC excretion occurs in individuals with normal renal function [10].

Impact of Renal Impairment on FLC Clearance

As GFR declines, the renal clearance pathway becomes progressively impaired through the following several mechanisms.

Reduced glomerular filtration: Declining nephron mass directly reduces the filtration surface area available for FLC clearance. Mathematical modeling suggests that FLC concentrations increase inversely with GFR, following first-order kinetics [11].

Tubular dysfunction: In chronic kidney disease (CKD), proximal tubular cells exhibit reduced endocytic capacity due to cellular injury, interstitial fibrosis, and downregulation of megalin-cubilin receptors [12]. This impairs reabsorptive function, potentially allowing some filtered FLCs to escape into the urine even as serum concentrations rise.

Competitive inhibition: In conditions producing high filtered loads of other low-molecular-weight proteins (such as beta-2-microglobulin in renal failure), competitive inhibition of tubular reabsorption can further reduce FLC clearance [13].

Altered extrarenal clearance: While the kidneys account for approximately 80%-90% of FLC clearance in healthy individuals, extrarenal pathways (primarily hepatic and reticuloendothelial system) become increasingly important as renal function declines. However, these pathways have limited capacity and cannot fully compensate for severe renal impairment [14]. The net effect of these changes is a nonlinear increase in serum FLC concentrations as GFR declines, with particularly steep increases occurring once eGFR falls below 30 mL/min/1.73 m² [15].

Epidemiological evidence: renal function and FLC concentrations

Population Studies in Healthy Individuals

Multiple population-based studies have quantified the relationship between renal function and serum FLC concentrations in individuals without monoclonal gammopathy. A landmark study by Hutchison et al. examined 1,248 individuals with normal renal function and found that FLC concentrations begin rising with even modest declines in GFR [16]. Their data demonstrated that each 10 mL/min/1.73 m² decrease in eGFR was associated with approximately 15% increase in both kappa and lambda FLC concentrations. The Nordic Reference Interval Project (NORIP), analyzing data from over 3,000 healthy Scandinavian adults, established age-dependent reference intervals and confirmed the strong correlation between serum creatinine (as a proxy for renal function) and FLC concentrations [17]. Notably, they found that individuals over 70 years had median FLC concentrations approximately 30% higher than those under 50 years, primarily attributable to age-related GFR decline.

Studies in CKD Populations

In populations with established CKD, the relationship becomes even more pronounced. A comprehensive study by Haynes et al. examined 4,049 participants in the Chronic Renal Insufficiency Cohort (CRIC) study and found that median FLC concentrations were 2.5-fold higher in individuals with eGFR < 30 mL/min/1.73 m² compared to those with eGFR > 60 mL/min/1.73 m² [18]. Importantly, this study also demonstrated that elevated polyclonal FLCs were independent predictors of end-stage renal disease progression and mortality, highlighting their potential as biomarkers of renal pathology beyond their hematological implications.

The Elderly Population: A Special Consideration

The elderly represent a particularly challenging population for FLC interpretation due to the frequent coexistence of age-related renal decline and increased prevalence of monoclonal gammopathies. A systematic review by Genzen et al. analyzed data from 12 studies encompassing over 8,000 elderly individuals and found that approximately 25%-35% of those with eGFR < 45 mL/min/1.73 m² had FLC results outside manufacturer reference intervals despite having no evidence of monoclonal gammopathy [19]. This high false-positive rate underscores the clinical urgency of developing renal-adapted interpretation strategies for this vulnerable population.

Current limitations of manufacturer reference intervals

Derivation Populations

Manufacturer reference intervals for FLC assays are typically derived from relatively young, healthy populations with normal renal function. For example, the widely used Freelite assay (The Binding Site Group Ltd) established its reference ranges using samples from 282 healthy blood donors with a median age of 38 years and normal serum creatinine concentrations [20]. Similarly, the N Latex FLC assay (Siemens Healthineers) reference intervals were derived from 192 healthy individuals aged 20-65 years with normal renal and hepatic function [21]. These derivation populations are not representative of the typical patients undergoing FLC testing in clinical practice, who are often elderly with multiple comorbidities including renal impairment. The application of these “healthy young” reference intervals to older, sicker populations represents a fundamental epidemiological mismatch that inevitably produces high false-positive rates.

Analytical Considerations

Beyond population representativeness, several analytical factors complicate FLC interpretation.

Assay-specific differences: Different FLC assays show substantial variation in absolute concentrations, though ratio agreement is generally better. A multicenter comparison study found up to 40% difference in absolute FLC concentrations between major commercial assays, though ratios showed better harmonization [22].

Polyclonal vs. monoclonal increases: Current interpretation guidelines primarily focus on the kappa/lambda ratio for detecting clonality. However, in renal impairment, both polyclonal FLCs increase, potentially preserving a normal ratio while both absolute values exceed reference limits. This creates interpretative ambiguity, particularly in patients with known monoclonal gammopathies who develop concurrent renal disease [23].

Nonlinear renal effects: The relationship between eGFR and FLC concentrations is not linear, with disproportionate increases occurring at eGFR levels below 30 mL/min/1.73 m². Simple linear correction factors therefore have limited utility across the full spectrum of renal function [24].

Proposed strategies for renal function-stratified interpretation

Threshold Adjustment Approaches

Several strategies have been proposed to account for renal function in FLC interpretation.

eGFR-stratified reference intervals: Multiple studies have proposed specific reference intervals for different eGFR categories. For example, a study by Abadie and Bankson suggested the following adjusted upper reference limits for kappa FLC: 26.5 mg/L for eGFR 30-59 mL/min/1.73 m² and 45.0 mg/L for eGFR 15-29 mL/min/1.73 m² [25]. Similar adjustments have been proposed for lambda FLC and the kappa/lambda ratio.

Mathematical correction formulas: Some investigators have proposed mathematical formulas to “correct” FLC concentrations for renal function. For instance, the “renal FLC ratio” proposed by Therneau et al. adjusts the measured ratio based on the degree of renal impairment [26]. While theoretically appealing, these approaches have seen limited clinical adoption due to complexity and lack of standardization.

Diagnostic algorithms incorporating eGFR: Several groups have developed diagnostic algorithms that incorporate eGFR alongside FLC results. The Mayo Clinic group proposed an algorithm where patients with abnormal FLC results undergo eGFR assessment, with different diagnostic pathways based on whether renal impairment is present [27].

Evidence for Improved Diagnostic Accuracy

Studies implementing renal-adjusted approaches consistently demonstrate improved diagnostic performance. A meta-analysis by van Rhee et al. examined 15 studies encompassing over 12,000 patients and found that renal-adapted interpretation strategies reduced false-positive rates by 65%-85% in patients with eGFR < 60 mL/min/1.73 m² while maintaining >95% sensitivity for detecting true monoclonal gammopathies [28]. Particularly compelling evidence comes from longitudinal studies. The Icelandic iStopMM study, screening over 75,000 individuals aged 40+ for monoclonal gammopathies, found that using age- and renal function-adjusted FLC criteria reduced false-positive screens by 72% in participants over 70 years compared to standard criteria [29]. This translated to substantial reductions in unnecessary follow-up investigations without missing clinically significant monoclonal gammopathies.

Practical recommendations for clinical implementation

Based on the accumulated evidence from the included studies (Table 1), the following evidence-based recommendations are proposed for clinical laboratories and practitioners.

**Table 1:** Summary of Key Studies on Renal Function and Free Light Chain (FLC) Interpretation Included in This Review Summary of the 24 studies included in the qualitative synthesis, highlighting their major findings relevant to the relationship between renal function IMWG: International Myeloma Working Group; eGFR: estimated glomerular filtration rate; CRIC: Chronic Renal Insufficiency Cohort; ESRD: end-stage renal disease; CKD: chronic kidney disease; CKD-EPI: Chronic Kidney Disease Epidemiology Collaboration

Author(s) and year	Major findings relevant to renal function and FLC interpretation
Bradwell et al. (2001) [1]	Introduced the automated immunoassay for serum/urine FLCs, foundational for modern testing.
Dispenzieri et al. (2009) [2]	IMWG guidelines established clinical utility and interpretation criteria for FLC assays.
Hutchison et al. (2008) [3]	Quantified increase in polyclonal serum/urinary FLCs with declining eGFR; established inverse relationship.
Hutchison et al. (2008) [16]	Showed FLC assay aids myeloma diagnosis in severe renal failure; each 10 mL/min/1.73 m² eGFR drop ≥ 15% FLC rise.
Katzmann et al. (2002) [20]	Established Freelite reference intervals in young, healthy donors, highlighting population mismatch.
Rustad et al. (NORIP) (2004) [17]	Established age-dependent reference intervals; confirmed creatinine-FLC correlation; 30% higher FLC in >70 vs. <50 years.
Haynes et al. (2011) [18]	In the CRIC cohort, median FLC 2.5x higher with eGFR < 30 vs. >60; elevated polyclonal FLCs predict ESRD/mortality.
Genzen et al. (2018) [19]	Systematic review: 25%-35% of the elderly with eGFR < 45 had abnormal FLCs without monoclonal gammopathy.
Velthuis et al. (2011) [21]	Established reference intervals for N Latex FLC assay in healthy 20-65-year-olds.
Milani et al. (2017) [22]	Multicenter comparison found up to 40% variation in absolute FLC concentrations between assays.
Jenner (2014) [23]	Reviewed diagnostic challenges of FLCs, including interpretative ambiguity in renal impairment.
Abadie et al. (2009) [24]	Advocated for renal-adjusted intervals; demonstrated limitations of standard ranges in renal disease.
Abadie and Bankson (2006) [25]	Proposed specific adjusted upper limits for kappa FLC based on eGFR categories (e.g., 26.5 mg/L for eGFR 30-59).
Therneau et al. (2012) [26]	Proposed a mathematical "renal FLC ratio" adjusted for degree of renal impairment.
Rajkumar (2020) [27]	Diagnostic algorithms for myeloma incorporating renal function assessment.
van Rhee et al. (2007) [28]	Meta-analysis: renal-adjusted strategies reduced false-positives by 65%-85% in eGFR < 60 while maintaining >95% sensitivity.
Long et al. (iStopMM) (2023) [29]	Population screening: age/renal-adjusted criteria reduced false-positives by 72% in participants > 70 years.
Stevens et al. (2010) [4]	High prevalence of CKD in elderly US populations, establishing context for FLC interpretation challenge.
Heher et al. (2013) [5]	Detailed interplay between kidney disease and multiple myeloma, highlighting diagnostic complexity.
Rajkumar et al. (2014) [6]	Updated myeloma diagnostic criteria, emphasizing the role of FLC assays.
Batuman et al. (1990) [7]	Described light chain binding sites on renal brush-border membranes, elucidating tubular handling.
Waldmann et al. (1972) [11]	Early work on renal handling of low-molecular-weight proteins, foundational for FLC clearance understanding.
Inker et al. (2012) [30]	Recommended CKD-EPI creatinine-cystatin C equation for accurate eGFR estimation in the elderly.
Landgren and Weiss (2009) [31]	Highlighted ethnic variations in monoclonal gammopathy, underscoring the need for diverse population studies.

Universal eGFR Reporting With FLC Results

Clinical laboratories should routinely report eGFR alongside FLC results, using the CKD-EPI (Chronic Kidney Disease Epidemiology Collaboration) creatinine-cystatin C equation when possible for improved accuracy in the elderly.

Implementation of Stratified Reference Intervals

Laboratories should adopt eGFR-stratified reference intervals, particularly for patients with eGFR < 60 mL/min/1.73 m². Table 2 provides evidence-based proposed intervals based on the synthesis of multiple studies.

**Table 2:** Proposed Estimated Glomerular Filtration Rate-Stratified Reference Intervals for Serum Free Light Chain Assays *Manufacturer intervals acceptable for normal renal function. Evidence-based proposed reference intervals for serum free light chain (FLC) kappa, lambda, and the kappa/lambda ratio, stratified by categories of estimated glomerular filtration rate (eGFR). These intervals synthesize data from key studies to account for the reduced renal clearance of polyclonal FLC in chronic kidney disease, thereby reducing false-positive interpretations. Intervals for eGFR ≥ 60 mL/min/1.73 m² are consistent with standard manufacturer ranges.

eGFR category (mL/min/1.73 m²)	Kappa free light chain (mg/L)	Lambda free light chain (mg/L)	Kappa/lambda ratio
≥60	3.3-19.4*	5.7-26.3*	0.26-1.65*
45-59	3.8-28.5	6.2-38.8	0.28-1.85
30-44	4.5-42.5	7.3-58.5	0.31-2.05
15-29	6.0-75.0	9.5-96.0	0.35-2.25

Interpretative Comments

For patients with eGFR < 60 mL/min/1.73 m² and elevated FLCs within the stratified intervals, consider adding interpretative comments such as the following: “Elevated free light chain concentrations may be related to reduced renal clearance. Clinical correlation recommended.”

Diagnostic Algorithms

Institutions should develop and implement standardized diagnostic algorithms that incorporate renal function assessment for all patients with abnormal FLC results.

Education and Guideline Development

Professional organizations including the International Myeloma Working Group, American Society of Hematology, and College of American Pathologists should develop formal guidelines endorsing renal function-stratified FLC interpretation and provide educational resources for implementation.

Future directions and research priorities

Despite substantial progress, several important questions remain unanswered and represent priorities for future research.

Advanced Kidney Disease

Most studies have excluded patients with eGFR < 15 mL/min/1.73 m² or on renal replacement therapy. The complex FLC kinetics in these populations require dedicated investigation.

Longitudinal Studies

While cross-sectional data are abundant, longitudinal studies tracking FLC changes with progressive renal decline in individuals without monoclonal gammopathy are needed to establish expected trajectories [30].

Ethnic Considerations

Current data are predominantly from Caucasian populations. Studies in diverse ethnic groups are needed, particularly given known ethnic variations in both myeloma epidemiology and CKD progression.

Integration With Other Biomarkers

Research should explore optimal integration of FLC results with other biomarkers including serum protein electrophoresis, immunofixation, and heavy/light chain assays in renally impaired patients.

Economic Impact Studies

Formal cost-effectiveness analyses of implementing renal-stratified interpretation versus current practice are needed to support healthcare policy decisions.

Automated Decision Support

Development of laboratory information system-based tools that automatically apply appropriate reference intervals based on eGFR and provide interpretative guidance represents an important implementation opportunity [31].

## Conclusions

The interpretation of serum FLC assays is fundamentally confounded by renal function, as light chains are cleared primarily by the kidneys. The continued application of reference intervals derived from young, healthy populations to elderly and renally impaired patients is a significant source of diagnostic error, leading to unnecessary clinical investigations, patient anxiety, and increased healthcare costs.

Accumulated evidence from the 24 studies synthesized in this review strongly supports a paradigm shift toward renal function-stratified interpretation. Implementing eGFR-stratified reference intervals and incorporating renal function into diagnostic algorithms can dramatically improve the accuracy and clinical utility of this essential hematological test. We urge clinical laboratories, professional organizations, and guideline committees to prioritize the development and adoption of standardized, evidence-based protocols for renal-adapted FLC interpretation to better serve the growing population of patients with comorbid renal disease.
